# Shape Up or Ship Out: Can We Enhance Productivity in Coastal Aquaculture to Compete with Other Uses?

**DOI:** 10.1371/journal.pone.0115912

**Published:** 2014-12-29

**Authors:** Peggy Schrobback, Sean Pascoe, Louisa Coglan

**Affiliations:** 1 School of Economics and Finance, Queensland University of Technology, Brisbane, QLD, Australia; 2 CSIRO Oceans and Atmospheric Flagship, EcoSciences Precinct, Brisbane, QLD, Australia; Università di Genova, Italy

## Abstract

Coastal resources are coming under increasing pressure from competition between recreational, commercial and conservation uses. This is particularly so in coastal areas adjacent to major population centres. Given high recreational and conservation values in such areas, economic activities need to be highly efficient in order to persist. Management of these industries must therefore also encourage efficient production and full utilisation of the areas available. In order to achieve this, managers must first understand the level and drivers of productivity, and how these can be influenced. In this study, by way of illustration, the focus was on the Sydney rock oyster industry within Queensland's Moreton Bay, a multiple use marine park with high recreational and conservation value adjacent to Australia’s third largest city. Productivity of the oyster industry in Moreton Bay is currently low compared to historic levels, and management has an objective of reversing this trend. It is unclear whether this difference is due to oyster farmers’ business choices and personal characteristics or whether varying environmental conditions in the Moreton Bay limit the capacity of the oyster industry. These require different management responses in order to enhance productivity. The study examined different productivity measures of the oyster industry using data envelopment analysis (DEA) to determine where productivity gains can be made and by how much. The findings suggest that the industry is operating at a high level of capacity utilisation, but a low level of efficiency. The results also suggest that both demographic and environmental conditions affect technical efficiency in the Bay, with water characteristics improvements and appropriate training potentially providing the greatest benefits to the industry. Methods used in this study are transferable to other industries and provide a means by which coastal aquaculture may be managed to ensure it remains competitive with other uses of coastal resources.

## Introduction

Sustainable management of coastal resources is an important policy goal for all governments of countries with coastlines [1]. This is particularly so in coastal waters adjacent to major population centers, where recreational, conservation and economic uses of the areas often co-exist and compete for space. The latter is particularly vulnerable, as non-market values associated with recreational use (such as recreational fishing or scuba diving) and marine conservation are often high, requiring areas designated for commercial purposes to either be efficiently utilized or given over to the non-commercial activities. Increasingly, marine conservation spatial planning tools are considering the opportunity cost of commercial activities forgone when aiming to achieve conservation objectives [2], and commercial activities that are not operating fully efficiently are most likely to be first displaced. Conversely, where commercial industries are maintained within coastal waters, it is important that management of these industries ensures that they operate at their most productive level.

Ensuring sustainable use of marine resources in coastal waters requires an understanding of drivers of productivity. Productivity analysis is a well established technique in applied economics, and has been applied to a wide variety of industries [3]. In this study, by way of example to illustrate its use in coastal aquaculture, we focus on the potential to enhance productivity in the Sydney rock oyster (*Saccostrea glomerata*) (SRO) industry in Moreton Bay, Queensland. Much of the area of Moreton Bay is designated as a multiple use marine park, with some areas designated as no-take zones, some for recreational fishing only and others for low-impact commercial fishing and aquaculture. The Bay is adjacent to Brisbane, the capital city of Queensland and the third largest city in Australia. Much of the Brisbane population use the Bay area for recreational purposes, and recreational use value is believed to far exceed the value of commercial activities in the Bay [4], [5].

Oyster farming in Moreton Bay has been particularly challenged over the past decades, with current production well below historic levels. At one point, Moreton Bay was the largest oyster producing region in Australia, supplying the Sydney and Melbourne markets as well as Brisbane [6]. Overfishing, disease and changes in market conditions have all contributed to the decline in the Moreton Bay industry [6]. However, the area available for production (i.e., under commercial leases) and number of farmers is still one of largest of all estuaries along the Australian east coast, yet total production is relatively low in comparison to other areas [7], [8]. Roughly 17 per cent of all Australian SRO lease holders are located in Moreton Bay, yet they produce less than 2 per cent (by value) of Australian SRO production [7].

In response to this declining production and the opportunity cost this creates in terms of alternative activities in the Bay this creates, the Oyster Industry Management Plan for Moreton Bay Marine Park [9] includes the objectives of increasing production from the existing leases, to promote the commercial industry development and to improve the image of the industry. The related policy of non-productive oyster leases [10] includes a provision for minimum production levels in oyster leases and the requirement to “show cause” for non-productive farmers as to why they should retain their lease.

Increasing production from a given set of leases can only occur through increased efficiency and/or capacity utilisation. To determine the extent of any potential production increases, the existence and causes of any inefficiency and/or capacity underutilisation needs to be determined. It is unclear whether the current situation of the SRO industry in Moreton Bay is due to oyster farmers’ business choices, farmers’ personal characteristics or whether environmental conditions in the Moreton Bay limit the capacity of the oyster industry in this region.

Coastal fishery and aquaculture production can be influenced by the condition of coastal waters, which can be affected by human activities that occur along shorelines (e.g., increasing urbanisation, industrial development, and run-off from agriculture) [11]. Furthermore, the personal traits of fishermen (e.g., experience, educational level) can also have an effect on the resourceful operation of coastal fisheries. The understanding of the relationship between production factors (e.g., degree of government regulation, age of labour force, weather, and coastal water quality) and the productive performance of fisheries helps determine the extent and means by which productivity can be enhanced.

In this study, the productivity of the Moreton Bay SRO industry was assessed through measures such as technical efficiency, scale efficiency, allocative efficiency and capacity utilisation. Factors that drive these measures were also examined. These approaches are being increasingly applied in aquaculture as a means of providing information to policy makers on how to improve productivity in these industries [12]. The method was based on a two-stage analysis approach, with productivity measures derived in the first stage using multi-output data envelopment analysis (DEA). In the second stage of the analysis, the influence of oyster farmers’ personal characteristics and environmental conditions at different production sites on the derived efficiency and capacity scores was examined.

### Moreton Bay Sydney Rock Oyster Industry

**Figure 1:**
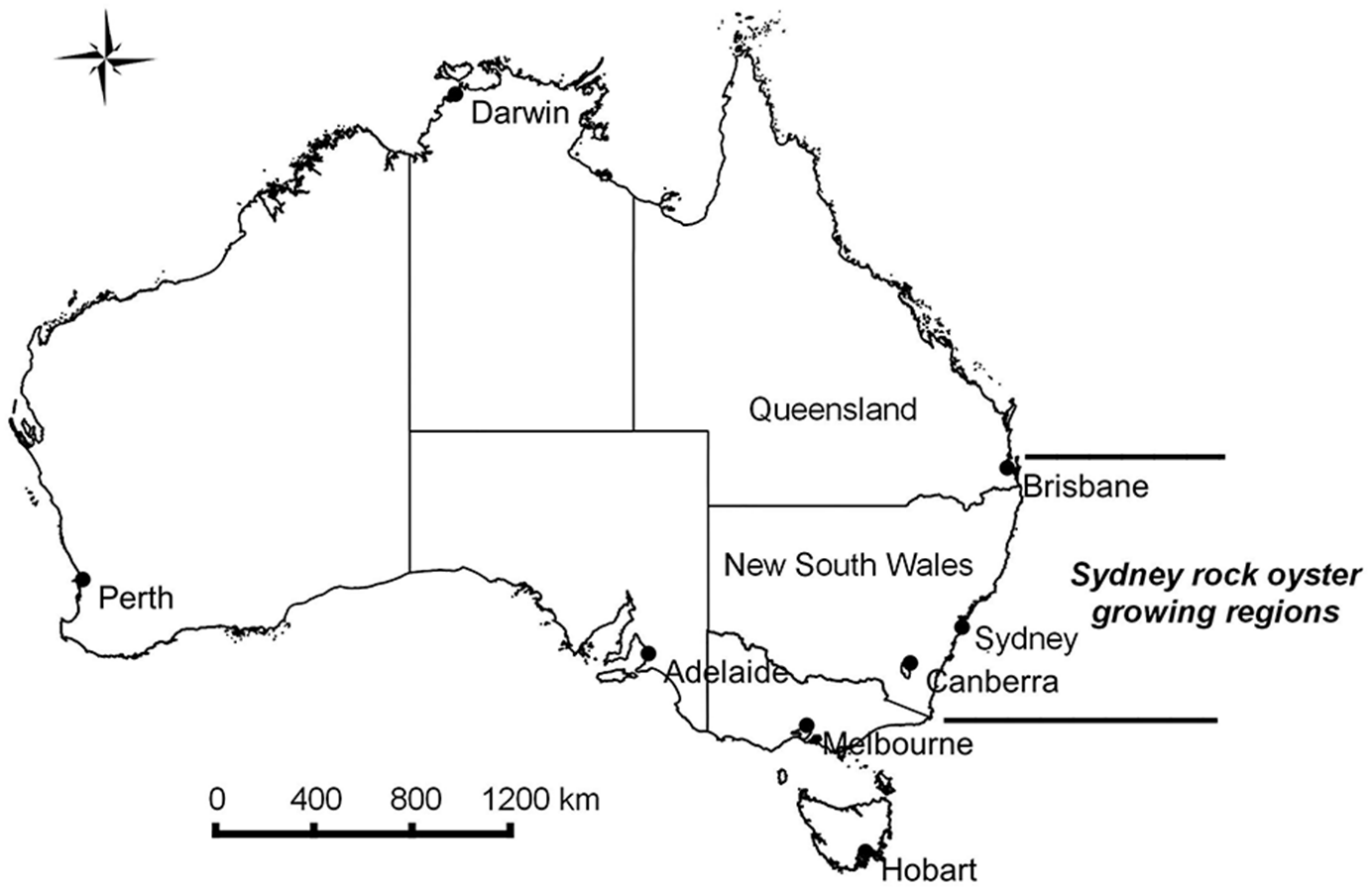
SRO growing regions in Australia.

The Sydney rock oyster industry which is one of Australia's oldest industries, dating back to European settlement [13]. This aquaculture industry is located in estuaries on Australia’s east coast ranging from the border between Victoria and New South Wales in the south to Moreton Bay in Queensland in the north (Fig. 1). The industry is based on a native species that naturally occurs in these waters. The SRO industry has been affected by a range of challenges over the past decades, including reoccurring disease outbreaks; the management of food security, biodiversity and environmental degradation risks; severe weather events; and market competition from the increasing production of the introduced Pacific oyster species [14].

The history of the SRO industry in Moreton Bay, the northern most cultivation area at the mouth of the Brisbane River, dates back to European settlement in Australia in the early 1800s [15]. The production of oysters in Moreton Bay peaked in the early 1900s [15], [16]. The decline in oyster production in Moreton Bay at that time was linked to mudworm infestation and severe depletion of natural oyster banks [6], [15], [16]. Since then oystering in Moreton Bay continued to be undertaken on a smaller scale in comparison to a relatively large industry in New South Wales. Currently there are 67 oyster farming businesses that take up a total of 97 approved leases in this estuary. In 2011–12 the total annual production volume of oysters in Moreton Bay was about 132,294 dozen valued at approximately $513,400 Australian Dollars [17]. The major market for SROs from the Moreton Bay is Brisbane, a metropolis with a population of 1.8 million people.

Moreton Bay is one of currently 65 Ramsar sites in Australia (which are unique wetlands that are of particular biological importance) [18] and the Bay is designated as a multiple use marine park. The Oyster Industry Management Plan provides an administrative framework for managing the oyster industry within the marine park. The plan is accredited under Marine Parks Regulations 2006 and oyster growers who conduct their operations within the framework of the plan do not require a marine parks permit.

**Figure 2:**
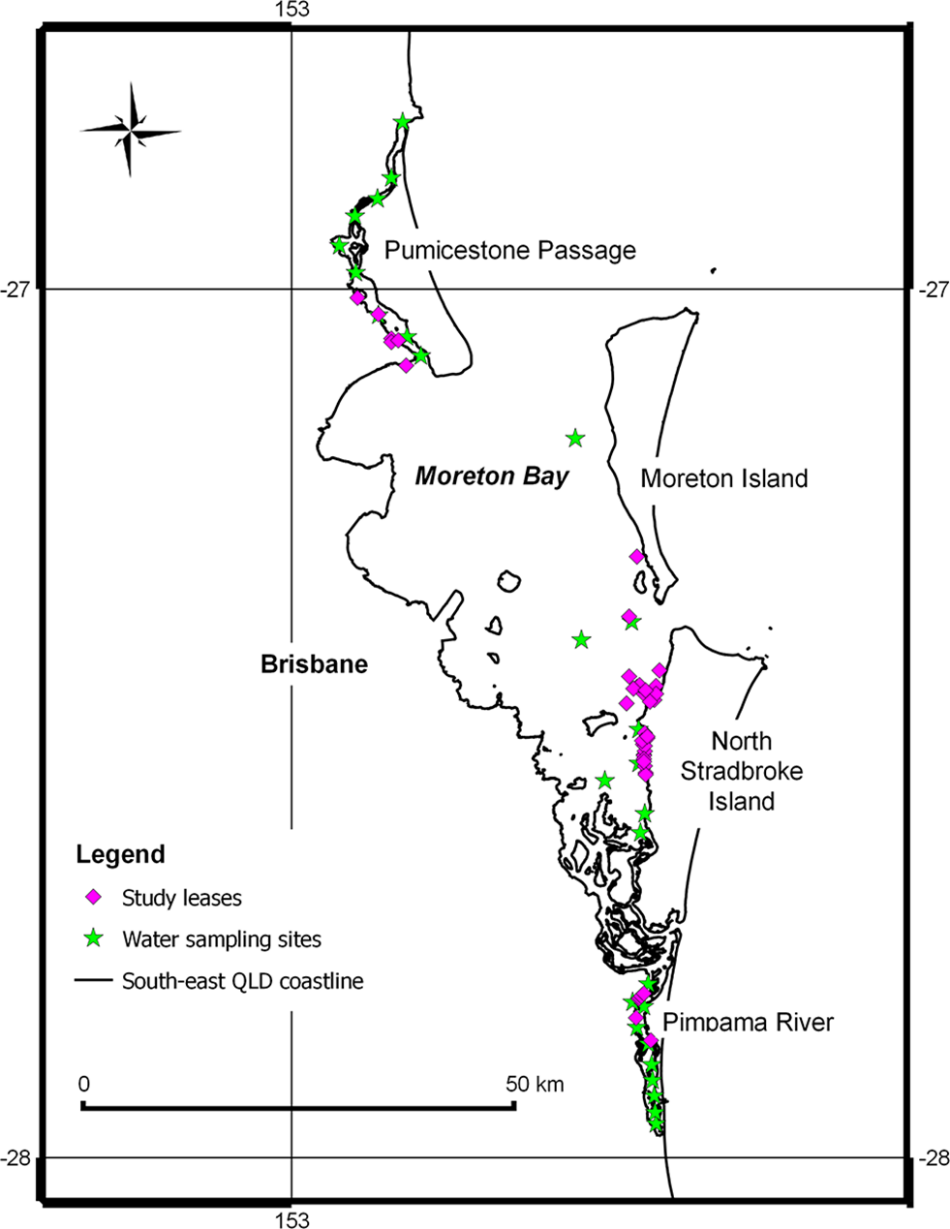
Moreton Bay oyster growing areas. Note: Moreton Island production area is referred to as Eastern Banks, North Stradbroke Island production area is referred to as Eastern Bay.

There are currently four areas allocated for oyster growing in Moreton Bay: Moreton Island (hereafter referred to as Eastern Banks), North Stradbroke Island (includes Myora and Canaipa, hereafter referred to as Eastern Bay), Pimpama River and Pumicestone Passage (Fig. 2).

The oyster areas are located within General Use, Habitat and Conservation zones of the Moreton Bay Marine Park [19]. Both (approved) commercial and recreational fishing activity can occur within oyster areas as long as the activity does not interfere with the aquaculture operation. The total area allocated to oyster leases covers about 435 hectares, which is less than one per cent of the total area of the marine park [19].

The Moreton Bay oyster industry is managed by the Queensland Department of Agriculture, Fisheries and Forestry (QLD DAFF). Resource allocation authorities issued under the Fisheries Act 1994 provide the holders the exclusive right to cultivate and to take oysters from the designated lease areas. Resource allocation authorities are issued for a period of up to 30 years.

SROs are filter feeding organisms and naturally occur in estuaries where the intertidal change provides a suitable habitat. This native Australian oyster species takes about 2.5 to 3.5 years to grow the smallest and largest marketable size, respectively. SROs are typically harvested in the warmer summer months ranging from October to March.

Being filter feeders, they can accumulate any type of pollution present in the water. The monitoring of the safety of oyster for human consumption includes regular water sampling at oyster areas and oyster meat sampling, with supply of oysters from particular leases stopped should water and meat samples not comply with food safety standards. Run-off from agricultural production in nearby river catchments can carry sediments into the estuary which may negatively affect water quality, oyster growth and also food safety. This is particularly problematic after high rainfall events, which also has the effect of reducing salinity further affecting the health of the oysters. The presence of high *E. coli* levels in meat samples, caused, for example, by sewage spills, is also observed occasionally, and leads to ceasing the supply of oysters from affected areas.

## Methods

In this study, a two-stage analysis procedure to analyse and assess inefficiency and capacity utilisation for two reasons was used. First, different producers harvest their oysters at different grow-out periods, resulting in a mix of size grades which requires a multi-output method of assessment. Second, anecdotal evidence suggested that there were a range of different approaches to production, ranging from effectively hobby farm to commercial enterprise. Statistical approaches, such as stochastic distance function approaches [20], [21], [22], [23], [24] effectively assume a common underlying production technology. The limited number of observations also makes parametric estimation of distance function models difficult. Consequently, data envelopment analysis (DEA) was undertaken in order to assess the level of efficiency and capacity utilisation for the Moreton Bay SRO industry. DEA is commonly applied in studies in the context of food production, fisheries and aquaculture [25], [26], [27], [28], [29], [30], [31] and is more commonly applied in general than parametric approaches for productivity analysis [3].

### DEA (first stage)

DEA is a non-paramedic, linear programming method for measuring the relative efficiencies of individual decision making units (DMUs) within a group of individual DMUs, given a set of inputs and produced outputs [32]. A DMU is a term that is frequently used in economics to refer to an individual or entity (e.g., firms, industries, countries) that are responsible for making production decisions [33], [34], [35]. In this study, the term DMU refers to individual oyster farmers who operate within the industry under given industry management plans and regulatory settings. Individual oyster farmers make decisions about how they will undertake oyster production, and that these decisions are reflected in the chosen production inputs and the obtained production outputs. For example, oyster farmers make decisions about the quantity and allocation of production inputs (e.g., labour input). Farmers also make decisions about the use of the total lease area (fixed inputs) they hold, and about their output production mix (e.g., harvest of small, medium or large sized oysters). The outcome of these decisions is the quantity harvested.

Given this notion, DEA can be used as a benchmarking tool to assess the performance of individual DMUs against the efficient frontier of the group which is defined by the most efficient DMUs within the group [36]. However, the frontier approach does not assume that most efficient DMUs within a group are fully efficient [36]; it rather provides a benchmark derived from observed efficient (or best-practice) DMUs.

A key feature of DEA is that it is readily able to incorporate multiple outputs into the analysis. This is particularly relevant since oysters are typically produced in three market sizes, that are small (bottle), medium (bistro) and large (plate). DEA does not impose any assumption about the functional form of the production function and thus is less prone to mis-specifications. However, as a non-parametric method, DEA cannot account for statistical noise and hence efficiency estimates may be biased if the production process is characterised by stochastic elements [36]. This is less problematic for capacity utilisation estimation, as the process for deriving unbiased estimates of capacity utilisation (shown below) has a benefit in that much of the effects of random error are cancelled out [37].

In this study, an output-oriented DEA model was used as the aim was to determine the maximum output of the *j*th DMU given observed inputs. The basic assumption of the output-oriented DEA is that output vector of the *j*th DMU is expanded radially until the combination of inputs of the respective DMU reached the efficient output frontier of the production possibility set for the group of DMUs. The form of the output-oriented DEA model can be given as:
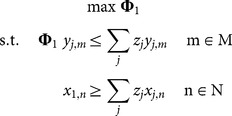
(1)where, Φ_1_ is a scalar indicating by how much the output of each DMU can be increased relative to the efficient frontier of a group of DMUs; *y_j,m_* is the amount of output *m* by DMU *j*; *x_j,n_* is the amount of input *n* used by DMU *j*; and *z_j_* are weighting factors. The input set can be separated into variable inputs (e.g., labour), where values of the variable may change in short-run and fixed inputs (e.g., the area of the lease), where values can only change in long-run. In order to account for the changes in the relationship between fixed inputs and outputs we can impose variable returns of scale (VRS), 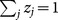 which allows for increasing, constant and decreasing returns within the production process. Various authors [38], [39] suggested the use of VRS in DEA models to account for situations such as imperfect competition and government regulation that may cause a firm to be unable to operate at optimal scale [36]. Without such a restriction, constant returns to scale (CRS) are assumed.

Technical efficiency (TE) is a measure that reflects the ability of DMUs to obtain maximum output from a given input set. The general form of TE is given by:
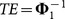
(2)


The value obtained for TE is the efficiency score for the *j*th DMU. The derived efficiency scores lie in the interval [0,1], with a value of 1 indicating a point on the frontier and hence a technically efficient firm.

Capacity represents the potential output given a set of fixed input, assuming that these are all fully utilised [40]. Most applied studies are concerned with the level of capacity utilisation, which measures the extent to which fixed inputs are being fully utilised [29], [31], [41], [42]. Capacity utilisation is derived by solving the above model (1) using fixed inputs only. The resultant technical efficiency measure, Φ_2_, can be used to derive a capacity utilisation score by:
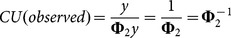
(3.1)


Färe et al. [42] argued that this CU measure may be biased downward, since it captures both capacity utilisation and technical efficiency. Consequently, an adjustment is required to separate out the CU component to correct for the bias. Färe et al. [42] suggest that an unbiased measure of CU may be calculated as:
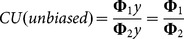
(3.2)


As noted above, this measure is also less susceptible to random error [37].

The scale efficiency measure provides information about the production scale or level of a DMU compared to other DMUs in a group. The CRS assumption is appropriate when DMUs are operating at an optimal scale [36]. However, the use of VRS imposes the possibility that the scale of production could affect the efficiency of DMUs. The scale efficiency measure is estimated as the ratio of technical efficiency with constant returns to scale (TE(CRS)) to technical efficiency with variable returns to scale (TE(VRS)), a TE(CRS) and a TE(VRS). The relationship can be described as:
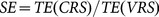
(4)


If the results for TE(CRS) and TE(VRS) scores for a DMU differ, it indicates that this DMU is operating at a scale that is less than efficient. Hence, the results provide an indication as to how close a DMU is to its (technically) optimal scale.

The allocative efficiency (AE) measures is used to identify the degree to which DMUs are adopting strategies that lead to optimisation of revenue from the production process, given the relative prices of each output. The estimation of the revenue efficiency with VRS and TE(VRS) are required for the estimation of allocative efficiency scores.

Revenue efficiency can be obtained by solving the following revenue maximization DEA problem:
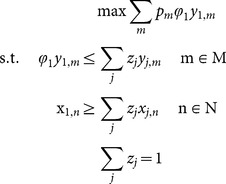
(5)


Where 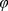 represents the linear expansion factor to the revenue frontier. The revenue efficiency is given by:
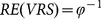
(6)


The output-mix allocative efficiency measure is then obtained as:
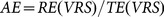
(7)


Allocative efficiency scores provide information about the degree to which changes in the production mix (i.e., production of small, medium, large and other sized oysters) could enhance the DMU’s revenues.

### Second Stage

The ability of DMU’s to convert input into outputs can be influenced by exogenous variables that characterise the environment in which production takes place [36]. These exogenous factors can be observable (e.g., government regulation, age of labour) and unforeseen (e.g., disease, weather) [36]. Previous studies that assessed the effect of drivers of efficiency derived in a DEA most commonly use Tobit analysis [29], [31], [43]. Efficiency scores, defined as ratios of actual output to the frontier value of the output, must lie between 0 and 1 or equal 0 or 1. Thus, the application of Tobit analysis is frequently used in the second stage analysis for [0,1] limited and censored distribution of the dependent variable [32], [44], [45]. Hoff [32] compares Tobit and OLS methods and shows that while the Tobit approach may be adequate, the OLS approach may in many cases replace Tobit as a sufficient second stage DEA model. McDonald [45] argues that DEA efficiency measures are not censored but rather fractional or normalized with a heteroskedastic distribution. In this case, Tobit analysis may produce mis-specified estimates, and OLS may be a more appropriate approach [45].

In this study, we apply both Tobit and OLS approaches to estimate how demographic characteristics and environmental conditions may affect productivity measures derived for oyster farmers in Moreton Bay.

### Data

Annual cross-sectional oyster production data were made available by the QLD DAFF. The initial production data set includes records covering the period between 1997/98 and 2011/12 for a total of 39 oyster farmers who gave the consent to DAFF for their data to be used for research purposes of this study. Since individual oyster farmers (the DMUs within the oyster industry) take decisions about their individual production process it can be assumed that these choices are reflected in the observed input and output production data.

The production data includes information about production output volume (number of dozens of oysters) and production values for four different product grades (sizes), namely bottle (small), bistro (medium), plate (large) and other. Product prices were derived from the production volume and values. In cases in which production values were unavailable for an individual, average annual prices derived from the available observation for each year were used.

The production data also included information on labour inputs, separated into three categories: lease owner full-time equivalent (FTE, based on 40 hour work per week), permanent FTE and casual FTE. Information about the total leased area size (hectare) per lease owner as well as the geographic location of the leases were used as fixed inputs. For larger scale oyster cultivation, there are commonly a number of leases used for different stage in the cultivation process. For example, the initial phase of catching of oysters spat usually requires areas where oyster spat is available in abundance while grow-out leases are used to fatten the oysters and depuration leases are used for purification prior to the harvest of oysters. Information collected from farmers that held more than one lease did not cover the particular use of each oyster leases during the cultivation process. Thus, the available production data only reported total production volumes and values per lease owner.

The total number of observations initially used for the first stage DEA was 288 (i.e., all observations). However, due to limitations in environmental and demographic data used in the second stage (see below), we also performed the first stage DEA on a sub-sample of 113 observations for which complete data were available. Descriptive statistics of the full and sub-sample of the data are shown in Table 1.

**Table 1:** Descriptive statistics of production data.

Variable	MeanFull-sample [sub-sample]	Coeff. of variationFull-sample [sub-sample]
*Outputs quantity (dozen)*		
Bottle grade	1,418 [1,925]	272% [201%]
Bistro grade	1,198 [1,314]	225% [222%]
Plate grade	612 [523]	221% [166%]
Other grade	513 [720]	288% [269%]
*Output price per dozen*		
Bottle grade	3.48 [3.87]	38% [32%]
Bistro grade	4.92 [5.69]	40% [39%]
Plate grade	6.57 [7.42]	37% [30%]
Other grade	3.94 [4.47]	58% [61%]
*Inputs*		
Hectare size	3.29 [10.23]	66% [188%]
Total labour (FTE)	0.13 [0.17]	218% [213%]

For the second stage analysis, we use demographic characteristics of oyster farmers and data of environmental parameters in proximity of the respective oyster leases. An oyster farm survey was undertaken by the authors of this study in 2012, which provided information about the socio-economic characteristics of Moreton Bay oyster farmers (see Table 2). The collection of this primary data set has been approved by the Queensland University of Technology’s Human Research Ethics Committee (approval number: 1200000303). The participants of the survey were made aware in written form about the confidential use of the data for research purposes and that the consent to use the data for this purpose is provided by the participants by completing the survey and returning it to the authors of this study. In order to comply with ethical research standards, none of the participants or their business are identifiable in this study.

**Table 2:** Socio-economic characteristics of the Moreton Bay oyster industry.

Socio-economic characteristics	Results	Socio-economic characteristics	Results
***Gender(per cent of all farmers)***		***Household***	
Female	17%	Number of people living in household	2.1
Male	83%	Number of children	2.2
***Age (years)***		***Annual income*** ** (weekly disposable income)^1,^** ^***#***^	
Minimum age	29	$0−$40,000 ($0−$669)	48%
Q1 age	51	$40,001−$60,000 ($670−$922)	5%
Average age	57.5	$60,001−$80,000 ($923−$1,174)	10%
Median age	56.5	$80,001−$100,000 ($1,175−$1,411)	29%
Q3 age	65	$100,001−$120,000 ($1,412−$1,646)	0%
Maximum age	76	Over $120,000 (over $1,646)	10%
Farmers younger than 35 years	4%	Off-farm income***^#^***	73%
***National origin^#^***		Proportion of total income from oyster farming (average)***^#^***	14%
Australian born	96%	**Other**	
***Experience in oystering (years)***		First Generation is oyster farming***^#^***	83%
Q1 experience	4	Average number of generation in oystering if not first generation	2.5
Minimum experience	0	Member in farming association***^#^***	100%
Q1 experience	4	Experience with other fish/shellfish species***^#^***	13%
Average experience	14		
Median experience	10		
Q3 experience	28		
Maximum experience	50		
***Educational attainment^#^***			
Year 10 certificate & below	30%		
Year 12 certificate	39%		
TAFE degree/Apprenticeship	4%		
University degree	26%		

Data collected in a farm survey in 2012. Weekly disposable income (net income) estimates for income brackets derived from Australian Taxation Office [71]. ^#^Per cent means, data represent as proportion on all farmers. All income values are in Australian Dollars.

The socio-economic characteristics of Moreton Bay oyster farmers were matched with the production data where available. The second stage analysis was undertaken on a sub-sample of the production data as demographic information was not available for all farmers. For observations that included demographic information, we augmented records of continuous variables (e.g., age, years of experience) to account for the continuous involvement of farmers in the industry. Categorical data (e.g., level of farmer education) was assumed to be constant over time, with dummy variables also included to capture any effects of gender (Male = 1), education (tertiary educational = 1) and generation (1 = more than one generations of experience in oyster farming within the family) on productivity.

Environmental data for Moreton Bay (see Table 3) were obtained from Healthy Waterways Ltd. [46]. This data set contains monthly records for water quality indicators collected at estuarine zones within Moreton Bay. The environmental data includes records ranging from 2000 to 2012. Although earlier records are available, they were collected by different agencies and contain less frequent and spatially distributed information and were therefore excluded from this analysis. We mapped oyster production areas against water collection sites and only used data for sites that were in close proximity to the production areas. Details are provided in Fig. 2.

**Table 3:** Environmental variables used in the analysis.

Environmental variable (unit)	Mean	Coeff. of variation
Salinity (ppt)	31.98	16%
Temperature (°C)	22.47	4%
Dissolved oxygen (%)	94.52	12%
Light penetration	3.34	57%
Turbidity (NTU)	5.92	116%
Dissolved total nitrogen (mg/L)	0.25	76%
Dissolved total phosphorus (mg/L)	0.02	52%
Chlorophyll-a (µg/L)	2.46	147%
pH	7.96	6%

Values refer to the zones Eastern Banks (sites 506, 507), Eastern Bay (sites 310–314, 502), Pimpama River (site 1801) and Broadwater (105–123) in the data set obtained from Healthy Waterways Ltd. [46] as they best represent areas in which oyster leases are located.

The key variables considered were salinity, water temperature, dissolved oxygen, light penetration, turbidity, dissolved nitrogen and phosphorus, chlorophyll-a levels, and acidity (pH). The relationship between oyster shell and flesh growth and environmental factors is very complex, depending on average as well as extreme levels and their duration.

Several of these variables are believed to have a direct impact on the growth and survival of the oysters. A low level of salinity may compromise the development and growth as oysters close their valves and stop feeding at low salinity levels [47]. Prolonged rainfall periods typically lead to low salinity levels in estuaries. Optimal salinity levels range from 25 to 35 parts per thousand (ppt) [48], [49]. However, the salinity tolerance varies significantly depending on the life stage of oysters, with younger oyster tolerating 15–39 ppt and adult oysters tolerating 0–50 ppt for limited period of time [49]. The optimal water temperature for SRO development and growth ranges from 14–28°C with a tolerance of 11–30°C [48], [49]. Low levels of dissolved oxygen also affect the metabolism of oysters [50]. High acidity in water affects shell formation of oysters and, thus, their growth [51], with the optimal pH range for SRO being 6.5–8.5 [52]. High level of turbidity, in particular of inorganic particles, may lead to congested gills affecting their ability to filter water and extract food [53]. Turbidity typically increases after rain events.

Other environmental variables affect the supply of the food source for oysters, indirectly affecting their growth. The depth to which light penetrates the water affects the presence of phytoplankton/microalgae biomass, an energy source for oysters. High level of turbidity also reduce the amount of light and therefore the food supply [53]. Similarly, a low level of oxygen may affect phytoplankton/microalgae biomass production. Chlorophyll-a is a direct measure of the presence of phytoplankton.

The level of dissolved nutrients can reduce the food safety of the oyster, with too high levels leading to harvesting being delayed. Nutrient levels also may affect the production of the food supply, with excessive levels leading to algal blooms, and in extreme cases eutrophication. Dissolved total nitrogen measures the presences of all forms of nitrogen (e.g., nitrate, nitrite, ammonia) in water. Urban and agricultural runoff, industrial wastes, and sewage effluents typically lead to high nitrogen concentrations in estuaries. Oysters are able to assimilate nitrogen from the water in their soft tissue and shells [54]. Dissolved total phosphorus is a measure for the presence of all forms of phosphorus present in water. The presence of high levels of phosphorous in estuaries can be attributed to similar sources as for nitrogen (see above). Oysters are able to assimilate phosphorus from the water in their soft tissue and shells [55].

Monthly records were used to obtain an annual average value for each environmental variable at each production area. Extreme values, such as annual minima or maxima, were not considered appropriate for our analysis as the data set does not provide information about the frequency and duration of extreme values within a month. Such information would have been vital for estimating the magnitude and significance of extreme environmental values on productivity [49], [52]. Since demographic observations were unavailable for leases in the Pumicestone Passage we could only include three for the four oyster production areas in Moreton Bay in the second stage analysis. Dummy variables for Eastern Bay and the Eastern Banks oyster growing areas (Fig. 2) were included to account for any spatial effects that are not picked up by environmental variables.

## Results

**Figure 3:**
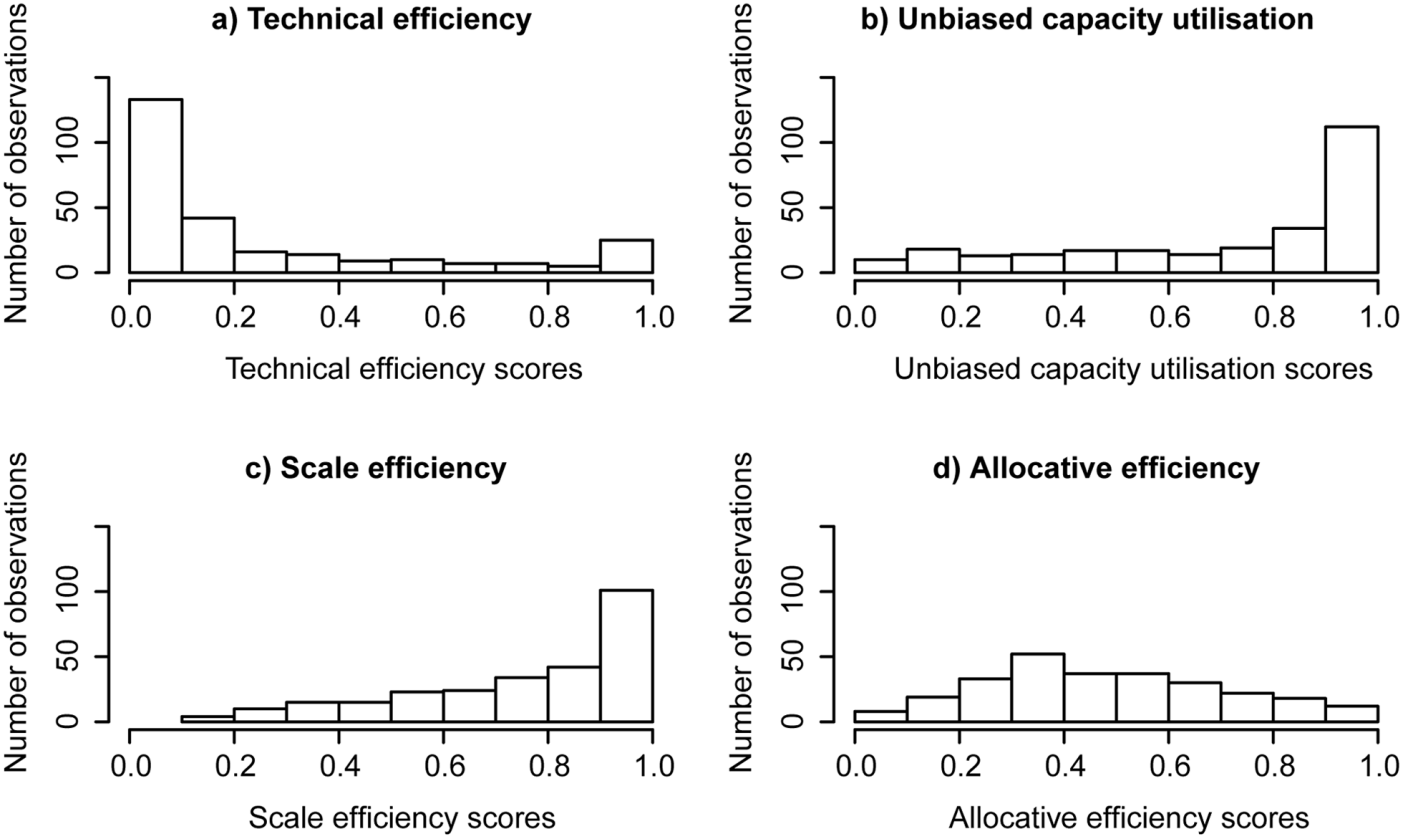
Distribution for capacity utilisation and efficiency scores (all observations).

The DEA analysis revealed that a high proportion of oyster businesses in Moreton Bay were relatively inefficient (Fig. 3.a, Table 4). In contrast, most of the oyster businesses operated at a high or full capacity utilisation rate (UCU median of 0.85, Table 4). That is, the businesses are mostly providing an appropriate amount of variable inputs (labour), but not are using it efficiently. The majority of oyster businesses operate close to or at the technical optimal scale (Fig. 3.c, Table 4) with a median scale efficiency scores of 0.81. Given this, we can conclude that most businesses in this industry would not be able to significantly increase their productivity by changing their level of activity (labour) or the scale of their operations.

**Table 4:** Summary of the key DEA results (all observations).

Capacity utilisation/efficiency measure	Min.	Median	Mean	Max.	Standarddeviation
Observed CU	0.000	0.059	0.177	1.000	0.267
Unbiased CU	0.018	0.850	0.716	1.000	0.305
TE (VRS)	0.000	0.099	0.249	1.000	0.311
Scale efficiency	0.104	0.808	0.751	1.000	0.232
Allocative efficiency	0.000	0.438	0.477	1.000	0.230

CU for capacity utilisation, TE (VRS) for technical efficiency (variable returns of scale).

Allocative efficiency scores were found to be relatively dispersed (Fig. 3.d, Table 4). Allocative efficiency compares technical efficiency against revenue efficiency and thus, indicates the degree of which changes in the output mix (different grades of oysters sold) could enhance the revenue of businesses in the industry. The wide distribution of allocative efficiency scores indicates that the current product mix is not optimal for a high proportion of the industry.

**Figure 4:**
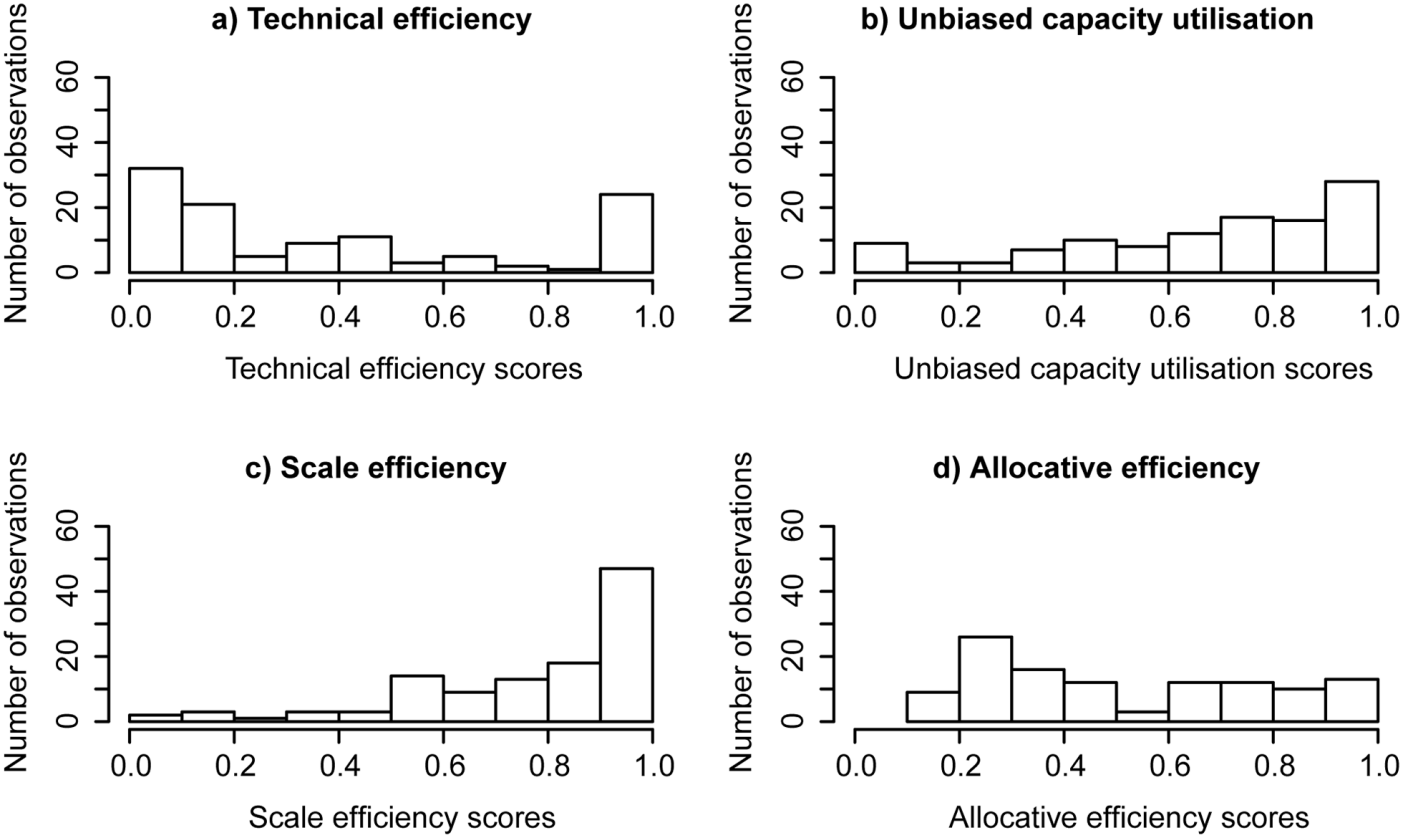
Distribution for capacity utilisation and efficiency scores (sub-sample).

The derived technical efficiency scores, capacity utilisation scores and scale efficiency scores for the sub-sample used in the first stage analysis follow a very similar distributional pattern with only minor variation in comparison to the results obtained in the analysis using the full data set (Fig. 4, Table 5). The distributions of allocative efficiency scores using the full data set and the sub-sample set show differing patterns (see Fig. 3.d, Fig. 4.d, Table 4, Table 5), which suggests that the results using the sub-sample data set should be interpreted with caution.

**Table 5:** Summary of the key DEA results (sub-sample).

Capacity utilisation/efficiency measure	Min.	Median	Mean	Max.	Standard deviation
Observed CU	0.000	0.114	0.262	1.000	0.313
Unbiased CU	0.010	0.752	0.664	1.000	0.293
TE (VRS)	0.001	0.291	0.398	1.000	0.367
Scale efficiency	0.038	0.847	0.778	1.000	1.000
Allocative efficiency	0.113	0.445	0.518	1.000	0.271

CU for capacity utilisation, TE (VRS) for technical efficiency (variable returns of scale).

The results for the second stage OLS and Tobit estimations are shown in Table 6 and Table 7. Both OLS and Tobit estimation methods generate consisted results with only minor differences in significance levels in TE and UCU model results. The TE and UCU models were jointly significant, although the explanatory power of the OLS models were generally low. While the allocative efficiency model was jointly significant using the Tobit approach, variables in the OLS model were not jointly significant. Thus, the second stage analysis results for the allocative model should be interpreted with caution.

**Table 6:** Tobit analysis results.

TOBIT	TE					UCU						Allocative				
Coefficients	Estim.	Std.Err.	p-val.	Magn.	Rank	Estim.	Std.Err.	p-val.	Magn.	Rank		Estim.	Std.Err.	p-val.	Magn.	Rank
(Intercept)	0.311	0.283	0.273			0.469	0.220	0.033				6.967	7.537	0.355		
Male	0.018	0.172	0.919	0.018		0.462	0.135	0.001	0.462	2		0.186	0.137	0.175	0.186	
Age	−0.291	0.059	0.000	0.291	5	−0.003	0.049	0.950	0.003			0.001	0.004	0.884	0.007	
Experience	0.141	0.044	0.001	0.141	8	0.016	0.035	0.654	0.016			−0.002	0.003	0.626	0.017	
Education_2	−0.219	0.114	0.055	0.219	6	−0.116	0.091	0.202	0.116			0.105	0.090	0.243	0.105	
Generation_2	−0.162	0.101	0.110	0.162		0.166	0.081	0.039	0.166	3		0.135	0.078	0.085	0.135	3
Salinity	0.019	0.058	0.749	0.019		−0.031	0.046	0.504	0.031			0.083	0.043	0.052	0.088	4
Temperature	0.057	0.041	0.172	0.057		0.009	0.033	0.793	0.009			−0.044	0.077	0.567	0.019	
Dissolved Oxygen	−0.008	0.063	0.897	0.008		−0.073	0.050	0.144	0.073			−0.017	0.021	0.421	0.039	
Light penetration	−0.307	0.107	0.004	0.307	4	0.068	0.082	0.412	0.068			−0.063	0.048	0.184	0.109	
Turbidity	0.592	0.172	0.001	0.592	1	−0.036	0.135	0.792	0.036			0.045	0.060	0.454	0.096	
Nitrogen	0.171	0.090	0.056	0.171	7	−0.010	0.072	0.889	0.010			−0.409	1.818	0.822	0.016	
Phosphorous	−0.351	0.134	0.009	0.351	3	−0.064	0.107	0.549	0.064			29.642	27.338	0.278	0.112	
Chlorophyll-a	−0.373	0.124	0.003	0.373	2	0.011	0.097	0.911	0.011			−0.150	0.186	0.419	0.075	
pH	0.128	0.068	0.062	0.128	9	−0.027	0.055	0.621	0.027			−0.881	0.880	0.317	0.053	
Eastern Banks	−0.290	0.454	0.522	0.290		0.657	0.359	0.067	0.657	1		0.773	0.348	0.026	0.773	1
Eastern Bay	0.301	0.307	0.328	0.301		−0.342	0.237	0.149	0.342			0.494	0.234	0.035	0.494	2
Log Sigma	−1.136	0.079	0.000			−1.368	0.075	0.000				−1.363	0.069	0.000		
**Model Statistics**	**TE**					**UCU**						**Allocative**				
Left-censored:	0					0						0				
Uncensored:	90					95						107				
Right-censored:	23					18						6				
Log-likelihood:	−46.663					−18.723						−13.128				
LR chi-squared:	71.116					62.660						23.081				
p-value:	0.000					0.000						0.000				

**Table 7:** OLS analysis results.

OLS	TE					UCU					Allocative				
Coefficients	Estim.	Std.Err.	p-val.	Magn.	Rank	Estim.	Std.Err.	p-val.	Magn.	Rank	Estim.	Std.Err.	p-val.	Magn.	Rank
(Intercept)	−14.467	8.402	0.088			4.368	7.078	0.539			5.517	7.715	0.476		
Male	0.020	0.153	0.894	0.020		0.458	0.129	0.001	0.458	1	0.207	0.141	0.144	0.207	
Age	−0.020	0.004	0.000	0.258	4	0.002	0.003	0.481	0.031		−0.000	0.004	0.994	0.000	
Experience	0.013	0.004	0.002	0.125	9	−0.001	0.003	0.819	0.008		−0.001	0.004	0.751	0.011	
Education_2	−0.195	0.102	0.058	0.195	6	−0.075	0.086	0.384	0.075		0.100	0.093	0.286	0.100	
Generation_2	−0.185	0.088	0.039	0.185	7	0.143	0.074	0.057	0.143	3	0.135	0.081	0.100	0.135	3
Salinity	0.017	0.047	0.728	0.018		−0.015	0.040	0.717	0.015		0.073	0.044	0.096	0.078	4
Temperature	0.109	0.086	0.211	0.046		−0.001	0.073	0.992	0.000		−0.044	0.079	0.578	0.019	
Dissolved Oxygen	−0.002	0.024	0.923	0.005		−0.018	0.020	0.381	0.040		−0.013	0.022	0.560	0.029	
Light penetration	−0.144	0.053	0.008	0.248	5	0.043	0.045	0.338	0.074		−0.052	0.049	0.293	0.089	
Turbidity	0.194	0.067	0.005	0.415	1	−0.016	0.057	0.777	0.034		0.034	0.062	0.582	0.073	
Nitrogen	4.122	2.050	0.047	0.159	8	0.150	1.727	0.931	0.006		−0.387	1.882	0.838	0.015	
Phosphorous	−76.796	30.719	0.014	0.290	2	−14.330	25.880	0.581	0.054		28.570	28.210	0.314	0.108	
Chlorophyll-a	−0.560	0.209	0.009	0.281	3	0.009	0.176	0.961	0.004		−0.126	0.192	0.513	0.063	
pH	1.751	0.981	0.078	0.105	10	−0.212	0.827	0.799	0.013		−0.714	0.901	0.430	0.043	
Eastern Banks	−0.407	0.389	0.297	0.407		0.540	0.327	0.102	0.540		0.700	0.357	0.053	0.700	1
Eastern Bay	0.170	0.261	0.515	0.170		−0.382	0.220	0.085	0.382	2	0.418	0.239	0.084	0.418	2
**Model Statistics**		**TE**				**UCU**					**Allocative**				
Df:		96				96					96				
Residual standard error:		0.289				0.243					0.265				
Multiple R-squared:		0.469				0.410					0.180				
Adjusted R-squared:		0.380				0.311					0.044				
F-statistic:		5.292				4.162					1.320				
p-value:		0.000				0.000					0.201				

The OLS estimation of the relationship between the assessed exogenous production factors and the derived TE scores suggest that the age of farmers has negative significant impact on TE scores (Table 7). Tertiary education, and two or more generations within families in oyster farming also had a negative effect on TE scores compared to farmers who have a lower educational level and are first generation in oyster farming. TE is likely to be positively influenced be a higher level of experience as an oyster farmer. Gender did not affect TE in our estimation.

Light penetration, turbidity, nitrogen, phosphorous, chlorophyll and pH were all found to be significant, as expected. The spatial dummy variables were not significant, suggesting any differences between areas were adequately captured by the environmental variables.

An OLS regression analysis using scaled independent variables provided information about the magnitude and rank of the impact that significant exogenous variables have on the TE score (Table 7). Based on this, most demographic and environmental conditions appear to affect the level technical efficiency of oyster businesses, with the latter having a generally greater influence.

In terms of (unbiased) capital utilisation, being male and of more than one family generation in the oyster business has a positive and significant impact. In contrast, none of the environmental variables had a significant effect on UCU. This is not surprising, as the measure reflects to a large extent the degree to which output could be increased by increasing variable inputs, all other things being equal. The dummy variables for Eastern Bay (in OLS model) and Eastern Banks (Tobit model only) were significant (Table 6 and Table 7), suggesting that output of leases in the Eastern Banks (positive coefficient) was more fully utilised than the other areas, while Eastern Bay (negative coefficient) had greatest potential to increase output from increased variable input use.

The results for the allocative efficiency models show that more than one generation in oyster business, average salinity and spatial dummy variables are weakly significant (Table 6 and Table 7). However, the F-statistic in the OLS model indicates that the variables are not jointly significant, and the very low R-squared coefficient suggest that these factors explain very little of the actual variation in allocative efficiency. Thus, we concluded that the level of allocative efficiency observed in the industry is likely explained by factors other than those assessed in this study.

We did not undertake a second stage analysis on the derived scale efficiency scores. Lease sizes are determined exogenously (by the Government), and are not within the control of the farmers.

## Discussion and Conclusion

Increasingly, marine protected areas are being designed to achieve ecological objectives with least cost in terms of forgone commercial production through allowing multiple uses of conservation areas [2], [56], [57]. However, with potentially a high opportunity cost in terms of conservation benefits at stake, it is essential that remaining commercial activities are managed as efficiently as possible. Conservation management in marine protected areas should therefore be complemented by effective fisheries and aquaculture management.

Ensuring that the productivity of economic activities is maximised requires an understanding of the potential output of the industry and the level and drivers of efficiency and capacity utilisation within the industry. In this study the case of the Moreton Bay SRO industry was considered as this is an industry with a long history in the region, but has declined substantially over recent decades. Further, it is based on a native species, so preservation of the industry also has conservation as well as commercial value. Finally, it is facing increasing competition from other activities within Moreton Bay for space, particularly recreational and conservation uses.

While production of the industry has declined, the number of active leases in the area, in contrast, has not decreased by the same proportion, suggesting substantial decreases in productivity in the region. The Oyster Industry Management Plan for Moreton Bay Marine Park [9] includes the objectives of increasing production from the existing leases, and measures are available in the related policy on management of non-productive oyster leases [10] to potentially confiscate leases that do not meet minimum performance standards.

Given a fixed number of leases, and given that these leases are mostly operated at the optimal scale (from the scale efficiency measures), production can only be increased through either working the leases harder or through increased efficiency. The distribution of capacity utilisation from the analysis suggests that the potential to increase output through greater utilisation is limited for many leases, although a small number of leases were relatively underutilised. In contrast, a high proportion of the leases were operating inefficiently, and improved efficiency is the only way in which total productivity is likely to increase.

The potential to increase efficiency in the area depends on the factors that drive inefficiency, and the degree to which these can be influenced by policy. From the second stage analysis, the key drivers of efficiency differences between farms were largely environmental, and largely related to water quality. Hence productivity improvements are more likely to be improved through improvements in water quality in the region than through activities of individual farmers. Declining water quality has been attributed to substantial degradation of other components of the Moreton Bay marine ecosystem [58], and there is an active program underway to improve water quality through improved catchment management [59], [60]. Oyster farming has not generally been associated with water quality impairment, with some studies suggesting both beneficial attributes (e.g., nutrient recycling) as well as negative aspects (e.g., oxygen consumption) [61]. An assessment of the effects on the water quality in Moreton Bay and subsequent effect on the productivity of the oyster industry was beyond the scope of this study.

Some farmer specific variables were found to be significant, however, some potential to improve efficiency (and hence production) does exist. The key farmer characteristics that affected the level of efficiency included age, experience, education and family history in the industry. As might be expected, efficiency decreases with the age of farmers but increases with their experience. The fishery is characterised by an older population, many of which enter the industry at a relatively old age (compared with most industries). When comparing these findings with more detailed information about socio-economic characteristics of oyster farmers collected in 2012 (shown in Table 1), we can conclude that there is a high degree of hobbyist or lifestyle oyster farmers present in this industry. This type of oyster farmers may have lower incentives in operating their business efficiently than commercial oriented farmers, and thus, this may explain the observed low technical efficiency.

In such a case, efficiency would be enhanced by recruiting younger farmers to the fishery with a greater dependence on the industry for income, but given generally low earnings from the activity and the higher opportunity cost of labour of younger (potential) farmers, due to the co-location with a major city, encouraging younger farmers to the industry is difficult. Given this, the potential requirement of minimum production volumes over a number of years may be counter-productive. While hobbyist or lifestyle farmers could be forced out of industry as a result of the policy, leases that subsequently became available may not necessarily be taken up by existing or new oyster farmers.

The efficiency increase associated with experience suggests that skills can be learnt through time which improves productivity. Understanding these skills and undertaking training may help expedite these productivity benefits. Experience is a common factor affecting efficiency in both wild caught fisheries [30], [62], [63] and aquaculture [43], [64], [65].

The result that higher levels of education do not necessarily increase efficiency (and may, in fact, decrease efficiency) is not uncommon in studies of aquacultural efficiency [66], [67], although other studies have found that efficiency levels are related to the level of education [68], [69]. In wild caught fisheries, Pascoe and Coglan [62] found that education improved the efficiency of vessels using mobile gear (e.g., trawl), but decreased the efficiency of fishers using static gear (e.g., lobster pots). Oyster farming is a largely passive activity, as there is relatively little ongoing intervention required in their husbandry. One possible explanation then is that more educated farmers may be more prone to unnecessarily employing too much labour trying to improve production with less than proportional results. As many of the farmers came to the industry at a more advanced age, another possibility is that more educated farmers came from occupations that involved a very different skill set to those who were less educated.

The outcome for allocative efficiency scores implies that there is the potential for changes in the production mix to enhance production revenues (Fig. 3 and Fig. 4). However, there was no significant link between observable demographic and environmental factors and the allocative efficiency scores. Key factors that may influence the production mix are the risk of stock loss and the need to maintain of a cash flow. Although oyster farmers would potentially gain a higher price for selling plate (large) sized oysters, this would take a longer than harvesting a smaller size, as SRO take 2.5 and 3.5 years to grown to bistro and plate grade respectively. During this extra year, there is the risk of production loss through diseases, water pollution, extreme weather events or poaching (an ongoing problem in the industry). More risk averse farmers are likely to harvest a higher proportion of their stock earlier than what otherwise might be considered optimal [70]. Maintaining a cash flow during this period may also be important for farmers, especially those who do not receive a sufficient income from off-farm activities.

The aim of this study was to assess productivity measures such as technical efficiency, scale efficiency, allocative efficiency and capacity utilisation measure for the Moreton Bay SRO industry, and to determine the extent to which these measures could be influenced by management to enhance productivity in the industry. We found that there is a relatively low level of technical efficiency in the industry. Some of this can be explained by differences in environmental conditions in Moreton Bay. As such, improvements in water quality in the Bay may result in increased productivity in the industry. However, some demographic traits of the farmers are also significant drivers of efficiency. In particular, the high numbers of pre-retirement hobbyists present in this industry who potentially undertake their oyster business with a low incentive for technical efficient production, and also potentially with the wrong skill set to operate efficiently. Forcing these producers out of the industry through command and control measures (i.e., minimum production requirements) may not be effective in increasing productivity as there are few incentives for younger farmers to enter the industry. Developing appropriate training programs aimed at specific skills may be a more effective means of improving efficiency in the industry.

While the results of this study are not immediately transferable to other coastal aquaculture (or fishing) industries competing in a multi-use environment, the methods we have employed are readily transferable. Multiple use management of marine protected areas requires each of the uses to be optimised. This is particularly so in high population areas where use is correspondingly high, and the opportunity cost in terms of foregone recreational or conservation value from an underperforming commercial activity may be substantial.
